# Characterization of BCMA Expression in Circulating Rare Single Cells of Patients with Plasma Cell Neoplasms

**DOI:** 10.3390/ijms232113427

**Published:** 2022-11-03

**Authors:** Libere J. Ndacayisaba, Kate E. Rappard, Stephanie N. Shishido, Sonia M. Setayesh, Guilin Tang, Pei Lin, Nicholas Matsumoto, Ching-Ju Hsu, Rafael Nevarez, Carmen Ruiz Velasco, Amin Naghdloo, Eric Yang, Kevin Kelly, James Hicks, Jeremy Mason, Robert Z. Orlowski, Elisabet E. Manasanch, Peter Kuhn

**Affiliations:** 1 Convergent Science Institute in Cancer, Michelson Center for Convergent Bioscience, University of Southern California, Los Angeles, CA 90089, USA; 2 Programs in Biomedical and Biological Sciences, Keck School of Medicine, University of Southern California, Los Angeles, CA 90033, USA; 3 Department of Biological Sciences, Dornsife College of Letters, Arts, and Sciences, University of Southern California, Los Angeles, CA 90089, USA; 4 Department of Hematopathology, University of Texas MD Anderson Cancer Center, Houston, TX 77030, USA; 5 Department of Aerospace and Mechanical Engineering, Viterbi School of Engineering, University of Southern California, Los Angeles, CA 90089, USA; 6 Norris Comprehensive Cancer Center, Keck School of Medicine, University of Southern California, Los Angeles, CA 90033, USA; 7 Catherine & Joseph Aresty Department of Urology, Institute of Urology, Keck School of Medicine, University of Southern California, Los Angeles, CA 90033, USA; 8 Department of Lymphoma/Myeloma, Division of Cancer Medicine, University of Texas MD Anderson Cancer Center, Houston, TX 77030, USA; 9 Department of Biomedical Engineering, Viterbi School of Engineering, University of Southern California, Los Angeles, CA 90089, USA

**Keywords:** multiple myeloma, circulating plasma cells, morphogenomics, rare single cell, liquid biopsy, HDSCA, peripheral blood, bone marrow aspirate, multimodal data, BCMA

## Abstract

B-cell maturation antigen (BCMA), a key regulator of B-cell proliferation and survival, is highly expressed in almost all cases of plasma cell neoplasms and B-lymphoproliferative malignancies. BCMA is a robust biomarker of plasma cells and a therapeutic target with substantial clinical significance. However, the expression of BCMA in circulating tumor cells of patients with hematological malignancies has not been validated for the detection of circulating plasma and B cells. The application of BCMA as a biomarker in single-cell detection and profiling of circulating tumor cells in patients’ blood could enable early disease profiling and therapy response monitoring. Here, we report the development and validation of a slide-based immunofluorescence assay (i.e., CD138, BCMA, CD45, DAPI) for enrichment-free detection, quantification, and morphogenomic characterization of BCMA-expressing cells in patients (N = 9) with plasma cell neoplasms. Varying morphological subtypes of circulating BCMA-expressing cells were detected across the CD138(+/−) and CD45(+/−) compartments, representing candidate clonotypic post-germinal center B cells, plasmablasts, and both normal and malignant plasma cells. Genomic analysis by single-cell sequencing and correlation to clinical FISH cytogenetics provides validation, with data showing that patients across the different neoplastic states carry both normal and altered BCMA-expressing cells. Furthermore, altered cells harbor cytogenetic events detected by clinical FISH. The reported enrichment-free liquid biopsy approach has potential applications as a single-cell methodology for the early detection of BCMA+ B-lymphoid malignancies and in monitoring therapy response for patients undergoing anti-BCMA treatments.

## 1. Introduction

Of the millions of B cells produced daily in the germinal centers of peripheral lymphoid organs, only a few will mature, survive, and commit to differentiation into plasma cells (PCs). The survival and proliferation of these post-germinal center (GC) B cells relies primarily on a set of survival and growth molecules that include the tumor necrosis factor (TNF) B-cell maturation antigen (BCMA, aka CD269 or TN FRSF17). BCMA is a type III transmembrane receptor glycoprotein whose gene, located at the 16p13 chromosomal region, is primarily expressed in mature and terminally differentiated B lymphocytes [1,2,3]. Beyond surface expression, a perinuclear staining of BCMA (BCMAp) has also been described and suggested to play a putative role in antibody production [4,5]. BCMA functions as the primary TNF membrane receptor for the cognate ligands A proliferation-inducing ligand (APRIL) and B-cell activation factor of the TNF family (BAFF) in the core signaling pathways that regulate the growth, proliferation, and survival of differentiating B cells in both benign and malignant disease states [6].

**Figure 1:**
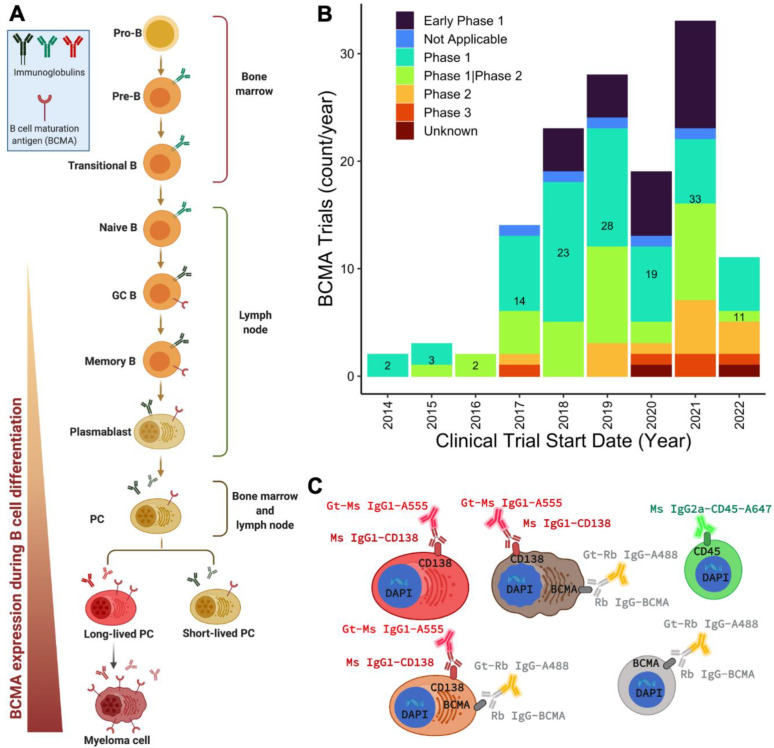
Assay rationale and Immunofluorescence targeting: (**A**) B-cell differentiation and BCMA expression during the B-cell maturation spectrum from GC B to mature plasma cells [7,8]. (**B**) Clinical studies involving BCMA as a therapeutic target of interest. Data from ClinicalTrials.gov (accessed on 1 May 2022) with “BCMA” as the search key term, as of April 2022. “Not Applicable” = study phase explicitly marked as “not applicable”. “Unknown” = no data entered for study phase. The low count in the year 2020 reflects the effect of the COVID-19 pandemic on clinical enrollment. (**C**) Immunofluorescence targeting with different antibodies for the detection of BCMA+ and CD138+ cells in the enrichment-free HDSCA workflow with histological features of plasma cells and B cells. Large CD138+ cells with eccentric nuclei represent the plasma cell compartment, while small CD45+ and BCMA+CD138- cells represent the B-cell fraction. Gt = goat, Ms = mouse, Rb = rabbit, Ig = immunoglobulin, A555 = Alexa Fluor 555 dye, A488 = Alexa Fluor PLUS 488 dye, A647 = Alexa Fluor 647 dye.

In lymphoproliferative cancers, BCMA is progressively expressed across the B-cell differentiation path, starting from late-stage B cells, proliferating plasmablasts, and PCs [3,7,8] (Figure 1A). In PC malignancies, BCMA functions primarily as a membrane receptor in key signaling cascades via NF-kB, MAPK/ERK, p38, and JNK/Elk-1 for the cell growth, proliferation, and survival of committed B lymphocytes and PCs [3,5], as well as in the maintenance of an immunosuppressive tumor microenvironment in multiple myeloma (MM) [7,9]. BCMA has consequently emerged as a promising diagnostic and prognostic marker of disease and a therapeutic target of interest in hematological malignancies—particularly in MM and its precursor neoplasms [10] (Phase I trial #: NCT05055063). As 80–100% of MM cell lines express BCMA [8,11] and nearly 100% of MM patients’ bone marrow (BM) expresses BCMA [7], the specificity in robust expression has motivated the development of multiple novel anti-BCMA immunotherapies that include chimeric antigen receptor T cells, bispecific antibodies, and antibody–drug conjugates, among other therapeutic modalities, some of which have achieved 90–100% clinical responses [7,11]. Since nearly all cases of MM relapse, quantifying BCMA expression would enable monitoring of therapy response and minimal residual disease. Additionally, detection and profiling of BCMA-expressing cells could be a method for monitoring minimal residual disease and finding therapy-resistant malignant B cells and PCs. Despite its critical role in disease pathogenesis and as a potent therapeutic target, surface BCMA has not previously been pursued and validated as a biomarker for routine clinical analysis outside of immunohistochemistry for core BM biopsy, which has been shown to be less sensitive than flow cytometry for the quantification of BCMA [12]. Previous research benchmarking BCMA against CD138—the current standard marker for PC identification—showed that BCMA is superior and more robust for isolating PCs in bone marrow aspirates (BMAs) [13]. Furthermore, there is limited literature on the expression of BCMA in the circulating cells of patients with hematological malignancies and, to the best of our knowledge, BCMA has not been used as a marker for the detection or isolation of circulating malignant PCs. A single-cell liquid biopsy method to robustly detect, morphologically characterize, and quantify circulating BCMA-expressing cells could establish BCMA as a key prognostic biomarker in both pre-malignant conditions and overt B-lymphoid cancers.

In this study, we report the technical development and initial genomic and clinical validation of a new slide-based, enrichment-free immunofluorescence assay (i.e., BCMA, CD138, CD45, DAPI) for the detection of circulating rare cells and morphogenomic profiling of BCMA+ cells in PC malignancies, hereafter referred to as the “BCMA assay”. The technical methodology is built on the established “no-cell-left-behind” approach of the high-definition single-cell assay (HDSCA) workflow—previously validated clinically for various pathologies, including breast cancer [14], myocardial infarction [15], melanoma [15], prostate cancer [16], bladder cancer [17], colorectal cancer [18], and multiple myeloma [19,20]—and has been optimized for the detection of BCMA expression for the characterization of MM precursor cells in PC neoplasia.

The BCMA assay was applied to patient samples collected from patients with MGUS (monoclonal gammopathy of undetermined significance), SMM (smoldering MM), NDMM (newly diagnosed MM), RRMM (relapsed/refractory MM), and LPL (lymphoplasmacytic leukemia). BCMA-expressing cells of varying size and morphology were detected, with BCMA and CD138 cell fractions characteristic of candidate normal and abnormal PCs, plasmablasts, and precursor myeloma cells. Downstream single-cell copy number variation (scCNV) analysis confirmed that the candidate aberrant cells detected harbor chromosomal genomic alterations also found using standard diagnostic clinical cytogenetics. The immunofluorescence liquid biopsy assay reported here for the identification and morphological delineation of circulating tumor cells in B-lymphoid malignancies has potential utility in the early detection of myeloma and monitoring of therapy response for patients undergoing anti-BCMA treatment.

## 2. Results

### 2.1. Assay Validation and BCMA Expression in Cell Lines and Spiked Normal Donor Samples

Technical validation for CD138, CD45, and DAPI cells in HDSCAs has been previously reported [19,20]. In titration experiments with U266, MM.1S, and control Jurkat cells, BCMA expression was consistently higher in U266 and MM.1S across all concentration conditions, while no BCMA expression was detected in control Jurkat cells, as expected (Figure S1A). Through these titration experiments, the final optimal concentration for the anti-BCMA antibody was determined to be 2.5 µg/mL when multiplexed with 2 µg/mL of anti-CD138 and 1.6 µg/mL of anti-CD45. To test BCMA specificity in circulating cells of normal donor (ND) controls, U266 cell lines were spiked in ND slides and stained with the four-color assay. In spiked-in stains, U266 cells were CD138+BCMA+CD45− and generally larger than surrounding white blood cells (WBCs) (CD138−BCMA−CD45+) with the canonical eccentric nucleus (Figure S1B), as expected, providing validation for the specificity of the anti-BCMA antibody for the assay. For assay sensitivity, linearity analysis on the serial dilution experiments shows the correlation between the number of U266 cells spiked in NDs and the total count of detected CD138+ (Figure S1C) and BCMA+ cells (Figure S1D). A significant positive correlation was observed, with R-squared values of 0.998 and 0.641, respectively. While only 0–5 CD138+ cells are expected to be found in unspiked ND samples, our experiments detected BCMA+ cells in ND samples (range: 5–110 cells), which were normal BCMA-expressing mature B cells. All of the BCMA+CD138− cells detected in the NDs were smaller than the U266 cells and equal in size to BCMA−CD138−CD45+ WBCs.

### 2.2. Characteristics of Patients in the Study Cohort

The patient cohort consisted of nine patients diagnosed with five different states on the PC neoplasia continuum, from MGUS to RRMM, with an additional patient with LPL and two age-matched NDs as controls. The cohort consisted of three males and six females with a median age of 63 years (range: 38–84). Clinical BM PC profiling showed higher malignant PC percentages consistent with myelomatous conditions except for MGUS2, who presented with 0% aberrant PCs. Accordingly, flow cytometry (FC) analysis found positive expression of disease markers (i.e., CD138, CD38, CD56) and negativity in normal/exclusionary markers (i.e., CD45, CD19). The LPL patient presented both PC and B-cell aberration with FC positivity in CD19 and CD45 in BM PCs, indicative of a histologically lymphoplasmacytic myeloma phenotype. Additional clinical features of the patients in this study are detailed in Table 1.

### 2.3. Morphological Characterization and Enumeration of BCMA+ Cells in Patients’ Peripheral Blood

**Figure 2:**
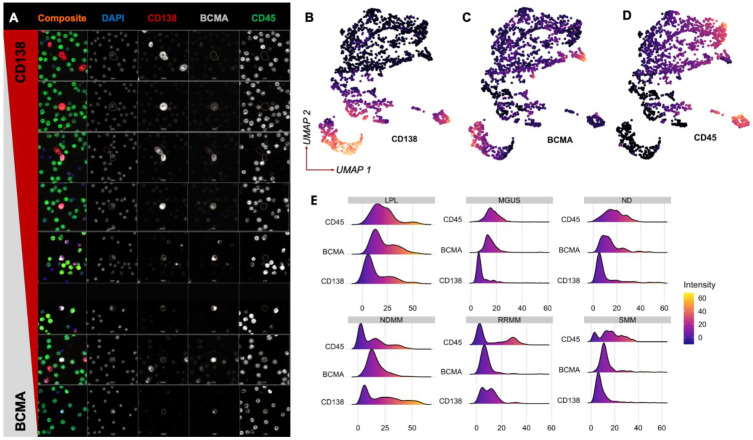
Morphological characterization of BCMA+ cells in PB samples: (**A**) Representative candidate rare cells with comparative BCMA and CD138 expression across different cell sizes. (**B**) UMAP projection of all candidate cells using all 761 single-cell morphology and marker intensity features, colored by CD138 expression. (**C**) UMAP projection, colored by BCMA expression. (**D**) UMAP projection, colored by CD45 expression. (**E**) Density plots showing channel intensities for each disease state.

Morphological analysis of cells in patients’ peripheral blood (PB) was performed to characterize the different detected rare cells and subsequent subtypes of BCMA+ cells. When comparing the expression of BCMA, CD138, and CD45 across all rare circulating cells, distinct patterns of expression were observed among rare cells of interest, with varying degrees of BCMA and CD138 expression across cell sizes and shapes (Figure 2A). We detected BCMA+CD138−CD45−, BCMA+CD138+CD45−, BCMA−CD138+CD45− and BCMA+CD138+CD45+ cells and characterized their varying cell size and morphology. The cellular subtypes represented candidate normal and abnormal PCs, plasmablasts, and precursor post-GC B cells. A staining pattern consistent with perinuclear BCMA (BCMAp) was also observed in patient microscopy imaging data (Figure S2), representing additional assay capabilities. Dimensionality reduction analysis by uniform manifold approximation and projection (UMAP) identified distinct groups, with two main clusters of BCMA+ cells, one of which was CD138+CD45− and the other of which was CD138−CD45+ (Figure 2B–D), with cell populations distinctly mapped in unique density distributions across CD138, BCMA, and CD45 intensities (Figure 2E).

**Figure 3:**
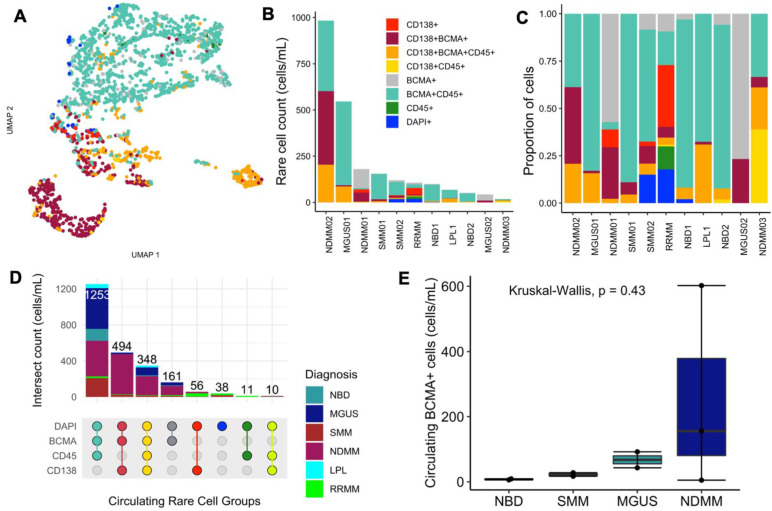
Quantitative enumeration of circulating rare and BCMA+ cells: (**A**) UMAP projection, colored by manual cell classification of rare cell groups. (**B**) The total cell count per immunofluorescence intensity classification. (**C**) Proportions of cells across patients. (**D**) Enumeration by channel type. The color codes in panels (**C**,**D**) are the same channel-type color codes in the legend shown in panel (**B**). (**E**) Total counts of BCMA+ cells across disease states. The boxplot colors are as shown in the diagnosis color scheme in panel D.

The staining intensities were consistent with manual classification of circulating rare cells, showing distinct populations of cells expressing CD138, BCMA, and CD45. The biomarker-based classified groups represented candidate PCs, committed clonotypic B cells, and plasmablasts (Figure 3A). Quantification of the different circulating rare cell groups across patients showed that CD138+ cells were more abundant in neoplastic myeloma and not in NDs, with a higher proportion being found in NDMM than in precursor states (SMM and MGUS) (Figure 3B–D), consistent with our prior observations [19,20]. BCMA+ cells were detected in all samples, including NDs, confirming that the expression of BCMA in early B cells, although suggested to be low, is detectable in our assay. Comparing the total BCMA+ count, BCMA+ cells were more abundant in disease states than in normal blood, with the highest total count observed in NDMM—the overt malignant state of NDMM (Figure 3E).

Together, the morphological analysis and enumeration in the assay shows that BCMA expression in circulating rare cells is found across different cell sizes and with varying CD138 and CD45 patterns, with higher counts in NDMM compared to other conditions.

### 2.4. scCNV for BCMA-Expressing Cells and Correlation with FISH Cytogenetics

**Figure 4:**
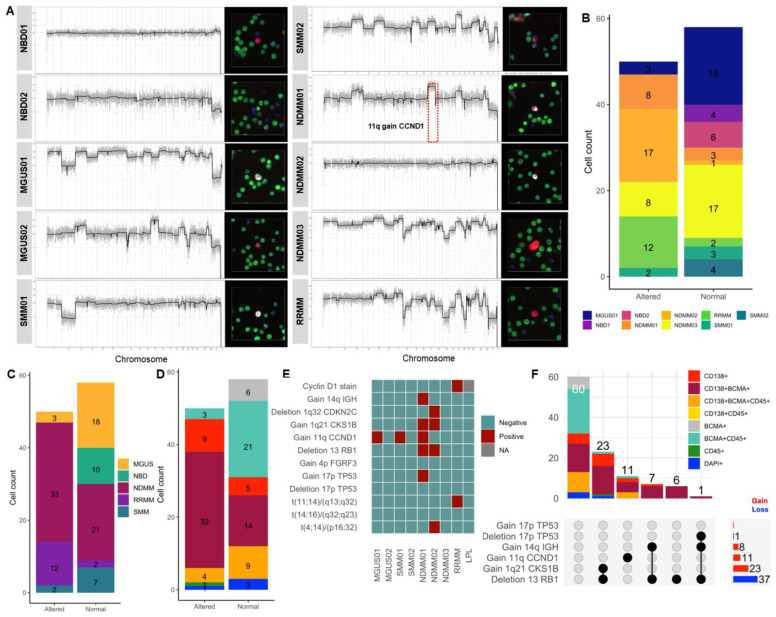
scCNV analysis for genomic validation of candidate aberrant cells: (**A**) Representative CNV profiles along with cellular morphology for each single cell. Dashed rectangles mark diagnostic cytogenetic events also detected in scCNV; red = gain, blue = loss. (**B**) Distribution of sequenced cell counts grouped by normal and altered scCNV profiles across patients. (**C**) Distribution of total counts of sequenced cells across diseases states (and NDs), grouped by normal and altered scCNV. (**D**) Distribution of total counts of sequenced cells across morphological groups, grouped by normal and altered scCNV. The color code is by channel type (as shown in panel (**F**)). (**E**) Clinical cytogenetic results from FISH and karyotyping for 12 common genomic events used for the diagnosis of myeloma. (**F**) Intersection plot showing total counts of altered single cells harboring alterations detected by clinical cytogenetics and corresponding morphological classification.

BCMA expression has been shown to be higher in malignant PCs than in normal PCs and is progressively expressed from post-GC B cells towards fully differentiated PCs [13]. To investigate whether circulating BCMA+ precursor B cells harbor genomic alterations, scCNV analysis of sequenced BCMA+ and BCMA− cells and correlation with FISH cytogenetic data were performed to validate the BCMA assay. Candidate circulating rare cells of interest from seven patients and two NDs were sequenced. No cells were picked and sequenced from the MGUS2 and LPL patients. Representative scCNV profiles and the respective marker expression and morphology of the sequenced single cells are shown in Figure 4A. Of all sequenced cells (*N* = 108), 50 and 58 displayed altered and non-altered CNV profiles, respectively. Quantitative cell counts for both normal and altered scCNV across patients (Figure 4B) and across disease states (Figure 4C) showed that altered cells were identified in all disease states and across 6/7 patients (the four sequenced cells in SMM2 had normal scCNV profiles). For controls, all cells from ND samples were normal (Figure 4B,C). Distribution of marker-based classification across normal and altered scCNV profiles is shown in Figure 4D with both normal and altered cells, covering the morphological classifications. The six BCMA+ cells detected had normal scCNV profiles. Only 3/24 (12.5%) BCMA+CD45+ cells were altered, while 32/46 (69.5%) CD138+BCMA+ cells were altered, 9/14 (64.3%) CD138+ cells were altered, 4/12 (33.3%) BCMA+CD138+CD45+ were altered, and only 1/3 (33.3%) DAPI+ cells were altered (Figure 4D).

For initial clinical validation, concordance analysis was performed for cytogenetic events across scCNV profiles. According to the clinical FISH cytogenetic results (Figure 4E), patients MGUS2, SMM2, NDMM3, and LPL were negative for the 12 key diagnostic cytogenetic targets, while MGUS1 and SMM1 were positive for only 11q (CCND1) gain. NDMM1 was positive for 17p (TP53) gain, 13 (RB1) deletion, 11q (CCND1) gain, 1q21 (CKS1B) gain, and 14q (IGH) gain. NDMM2 was positive for t(4;14)/(p16;32) translocation, 13 (RB1) and 1q32 (CDKN2C) deletions, and 1q21 (CKS1B) gain. RRMM was positive for t(11;14)/(p13;q32) translocation and cyclin D1. The positive FISH cytogenetic events, which are detected in CD138+ BM PCs, were mapped onto the scCNV profiles of circulating rare cells with morphological subtypes as classified by the BCMA assay detection; 48/50 (96%) of the altered cells, spanning five morphological groups, harbored at least one of the positive cytogenetic events (Figure 4F). The CD138+BCMA+CD45− morphotype constituted the most altered group, with cells spanning across six FISH cytogenetic events.

Taken together, the genomic data point to heterogeneous BCMA expression across normal and altered PCs, as well as the precursor plasmablasts and post-GC B cells. Altered CNV profiles were predominantly found in the CD138+BCMA+ phenotype, consistent with prior observations that BCMA has higher expression specific to PCs [8]. BCMA+CD45+ constituted a primarily unaltered fraction of cells that were likely normal BCMA+ post-GC and memory B cells.

## 3. Discussion

BCMA is a TNF receptor in the NF-κB, MAPK/ERK, p38, and JNK/Elk-1 signaling pathways that promotes cells’ growth, proliferation, and survival, as well as maintenance of an immunosuppressive tumor microenvironment in both benign and neoplastic myeloma and other lymphoproliferative malignancies [7]. Detection and characterization of circulating PCs and their precursor plasmablasts and B cells early in the disease progression, as well as in relapsed patients, remain a critical challenge due to the low abundance of cell populations of interest (rare cell context), such as clonotypic B cells and plasmablasts in early MGUS or therapy-resistant clones in relapsed conditions [7,11]. The exclusivity and robustness of BCMA expression in myeloma renders it a key and reliable marker for the identification of malignant PCs [13] and an exciting target for various therapeutic modalities [11].

This study reports a new immunofluorescence assay (i.e., CD138, BCMA, CD45, DAPI) for enrichment-free detection and characterization of BCMA-expressing cells in PC neoplasms, along with initial validation for utilizing this technology for tracking these cells across the myeloma progression spectrum. The primary aim of the report assay was to detect and characterize precursors of malignant PCs in lymphoid cancers. While CD138 and CD45 are standard markers in PC characterization and clinical diagnosis, BCMA is a newly described clonal B-cell and PC marker of great interest in hematological malignancies [7,10], conferring this assay with broader capability for detecting and characterizing terminal PCs, plasmablasts, and post-GC B cells. Furthermore, the BCMA assay identifies cells expressing both the cell-surface transmembrane BCMA and the perinuclear Golgi BCMAp [4], potentially extending the assay’s capabilities.

With BCMA as the cornerstone marker and in combination with CD138, the assay is an ideal rare cell technology capable of simultaneously detecting and characterizing clonotypic B cells; plasmablasts; normal, malignant, and dendritic PCs; and other non-canonical BCMA-expressing cells, with potential impact in detecting myeloma cells in MGUS and SMM. The BCMA+CD45+ cells in ND samples, confirmed to be genomically normal, are hypothesized to be normal activated post-GC B cells with a high proliferation index, likely due to their active maturation towards terminally differentiated PCs.

Among the notable limitations in this work is the small cohort size (*N* = 9) spanning different disease states. Future studies on a larger cohort could provide further insights into the patterns of BCMA expression in the PB of patients with hematological malignancies. Furthermore, myeloma patients present a serum-soluble BCMA (sBCMA), resulting from the shedding of membranous BCMA cleaved from PCs by gamma-secretase [22]. sBCMA predicts anti-BCMA therapy response and progression-free survival (PFS) in RRMM [23,24], as gamma-secretase inhibition improves anti-BCMA immunotherapy efficacy [25]. It has been recently shown that increased sBCMA in serum is correlated with poor prognosis in MGUS and SMM [24]. While we observed nucleus-free BCMA+ events in our staining, further characterization of sBCMA is needed to understand the spectrum of BCMA, but this was beyond the scope of this initial analysis. Additionally, multiplexed proteomic profiling for candidate post-GC B cells, plasmablasts, and dendritic PCs is warranted for further validation of cell immunophenotypes beyond the four markers in this assay, in order to assign the distinct phenotypes that characterize these candidate precursor cells. For therapy response monitoring, the BCMA assay has not yet been validated in patients receiving BCMA-targeted therapies with agents such as ADCs and bispecific antibodies that may potentially cause BCMA internalization or sterically hinder antibody binding in the assay. As such, additional validation in such a cohort of patients, with longitudinal single-cell monitoring, could elucidate any potential false negatives in the assay.

With ultra-rare cell detection capabilities and clinical validation, this assay has multiple applications in basic research and clinical care. Beyond the detection and characterization of BCMA+ cells, another area of interest is in the search for myeloma stem cells (MMSCs)—a rare cell population thought to be quiescent myeloma cells that behave as tumor-initiating cells as a result of their interaction with the tumor microenvironment [26]. MMSCs have been hypothesized to be a subgroup of plasma B-cell precursors that are CD24+ [27] and/or have a plasmablast phenotype with CD24+ expression [27]. We believe that these cells are among the BCMA+ cells detected by this assay, and further molecular characterization will provide additional evidence.

Prospective studies with large cohorts incorporating patients undergoing anti-BCMA therapies will provide additional validation for the clinical utility of this assay for early disease detection and monitoring of MRD, toxicity, and therapy response to anti-BCMA treatment modalities. Furthermore, with the versatility of the HDSCA platform, newly identified targets of interest such as GPRC5D [28,29], FcRH5 [30,31], CD73 (5′-nucletidase) [32], and others [33] can be adapted into the immunofluorescence technology for custom targeted cell-based measurement of disease and therapy response for treatment agents targeting these proteins in both preclinical and clinical development. Finally, multiplexing to six or more channels would allow for further clonotypic characterization of the precursor cells. Incorporation of CD19 or CD20 for normal B cells and CyIg*Kappa* or CyIg*Lambda* for light-chain monoclonality would enable higher precision and accuracy in detecting ultra-rare single cells driving the initiation and progression of lymphoid cancers.

## 4. Materials and Methods

### 4.1. Patient Enrollment and Sample Acquisition

Following an institutional review board (IRB)-approved protocol (PA18-1073), patients enrolled in this study provided informed consent and were accrued at the University of Texas MD Anderson Cancer Center (MDACC; Houston, TX, USA). For standard diagnostic workup, patients underwent a BM core needle biopsy, bloodwork, 24 h urine collection, and whole-body imaging. For this study, PB specimens from the diagnostic draw were collected from nine patients (two MGUS, two SMM, three NDMM, one RRMM, and one LPL) who were prospectively enrolled between 10 April 2019 and 9 February 2021. For each patient’s draw, a second tube was analyzed via FC at MDACC as part of the standard MM diagnostic workup. Two ND blood samples from individuals with no previously known pathologies were procured from the Scripps Clinic Normal Blood Donor Service (La Jolla, CA, USA). PB specimens were drawn and collected using anti-coagulated preservative tubes (Cell-Free DNA blood collection tube, Streck, La Vista, NE, USA), shipped to the USC Michelson Convergent Science Institute in Cancer (CSI-Cancer) via FedEx overnight, and processed as previously described using the established and validated HDSCA workflow [34]. Briefly, the samples underwent red blood cell lysis using an isotonic ammonium chloride buffer, and all nucleated cells were plated as a monolayer onto custom-made glass slides (Marienfeld, Germany) at approximately 3 million cells per slide. Plated slides were cryopreserved for future staining experiments and analysis.

### 4.2. Fluorescent In Situ Hybridization (FISH) and Karyotyping for Clinical Diagnosis

Following the diagnostic workup protocol for monoclonal gammopathies, standard FISH was performed for patients in this cohort. To increase sensitivity for the detection of cytogenetic abnormalities in PC neoplasms, enrichment of CD138+ cells using RoboSep-STM (Stem Cell Technologies, Vancouver, BC, Canada) for PC selection was performed on BMA collected at the time of study enrollment. The MDACC Cytogenetics Laboratory developed and evaluated the performance characteristics of the tests following the requirements established by the CLIA’88 regulations. To successfully perform FISH analysis for myeloma-associated aberrations, the clinical laboratory requires that the proportion of neoplastic PCs be more than 0.05% of the total cells analyzed, while aberrant neoplastic PCs must represent more than 25% of all PCs as analyzed by flow cytometry. For karyotyping, metaphase cells were prepared from BMA specimens cultured without mitogens for 24 h or with lipopolysaccharide (LPS) for 72 h, and chromosomal analysis was performed using standard techniques. The results were reported according to the International System for Human Cytogenetic Nomenclature (ISCN 2020) from 20 Giemsa-banded metaphases. Following the manufacturer’s specifications (Abbott Molecular, Abbott Park, IL, USA) for FISH detection of chromosomal rearrangements in t(4;14)/IGH::FGFR3, t(11;14)/IGH::CCND1, as well as copy number changes of CDKN2C/CKS1B, RB1/13q34, and TP53/CEP17 in PC myeloma, FISH analysis was performed on interphase nuclei acquired from BM cells utilizing dual-color FISH probes targeting the aforementioned common myeloma abnormalities. For each probe, analysis was performed on 200 nuclei, and the results were reported according to threshold values as previously established by the MDACC’s clinical cytogenetics laboratory.

### 4.3. Selection of Markers and Immunofluorescence Targeting for Assay Development

The rationale for developing the BCMA assay is grounded in BCMA’s progressive expression across the B-cell differentiation spectrum (Figure 1A) and the increasing interest in this marker as a therapeutic target, as demonstrated by the rise in clinical studies over the past 9 years (Figure 1B). The selection criteria for CD138, CD45, and DAPI have been previously described in two validated myeloma assays for morphogenomic differentiation of malignant and normal PCs and identification of rare cell clones [19,20]. Here, we substituted CD56 with BCMA for a new immunofluorescence staining panel (CD138, BCMA, CD45, DAPI), with the primary goal of targeting key cells of interest (Figure 1C) and characterizing circulating B lymphocytes expressing BCMA in the PB of patients with PC neoplasms. While a CD56-based assay [20] focuses on discriminating normal from malignant PCs and provides key insights for our ability to detect and characterize rare single cells in lymphoproliferative cancers, the BCMA assay’s prime goal is the detection of the precursors of the CD56+CD138+ (malignant plasma phenotype) cells. While the underlying technology is similar, the different markers differentiate the assays. 

For assay development and validation, mouse anti-human IgG1 CD138 (Exbio, clone A-38, cat# 10-520-C100, Vestec, Czech Republic), rabbit anti-human IgG BCMA (Abcam, cat# ab253242), and directly conjugated mouse anti-human CD45-Alexa647 (AbD Serotec, cat# MCA87A647, Raleigh, NC, USA) were acquired. Additionally, goat anti-mouse Alexa Fluor® 555 (cat# A21127, Invitrogen, Waltham, MA, USA) and goat anti-rabbit Alexa FluorPLUS® 488 (cat# A32731, Invitrogen, Waltham, MA) were procured as secondary antibodies targeting CD138 and BCMA, respectively.

### 4.4. BCMA Staining and Validation in Cell Lines and Spiked ND Samples

Anti-CD138 and anti-CD45 antibody testing and validation were performed as previously described [19,20]. BCMA testing was performed on U266, MM.1S, and Jurkat cell lines. An immunoglobulin E lambda myeloma-derived U266 cell line (gift from Dr. Akil Merchant; U266B1 TIB-196TM), an immunoglobulin A lambda myeloma-derived MM.1S cell line (ATCC® CRL-2974™), and a T-lymphocyte-derived Jurkat cell line (ATCC® TIB-152™) were cultured according to the manufacturer’s specifications. Slides for assay development were generated with pure cell lines (i.e., U266, MM.1S, and Jurkat) to test the expression of BCMA in myeloma and control cell lines. To establish the optimal concentration for anti-BCMA, we performed antibody titrations from concentrations of 0 to 10 μg/mL using contrived samples with the U266, MM.1S, and Jurkat cell lines.

For specificity of the anti-BCMA antibody, Jurkat cells were used as a BCMA− control. For specificity of the mouse anti-human Alexa Fluor® 555 secondary antibody, a no-primary-antibody control in which the anti-BCMA antibody was omitted from staining was used. To analyze the expression of BCMA in cell lines compared to normal WBCs, the U266 cell line was spiked into ND blood at a 1:100 dilution. For assay sensitivity testing, ND slides were spiked with U266 cells in serial dilutions of 0, 1, 10, and 100 cells per ND slide. Spiked slides were stained using the final assay concentrations and conditions (Table 2), and the numbers of detected CD138+ and BCMA+ cells were correlated with the spiked cell line counts for linearity analysis.

### 4.5. BCMA Staining and Validation in Patients’ PB

For the detection of BCMA-expressing cells in patients with PC neoplasia, PB slides were stained with the developed assay in patients with MGUS, SMM, NDMM, RRMM, and LPL. Slides were thawed after storage at −80 °C, fixed with 2% paraformaldehyde for 20 min, and washed using TBS. The slides were incubated with 10% goat serum for 30 min to block non-specific binding sites for secondary antibodies. Next, the slides were incubated with a primary antibody mix consisting of CD138 (2 µg/mL, Exbio), CD45-Alexa647 (1.6 µg/mL, AbD Serotec), and BCMA (2.5 µg/mL, Abcam) for 1 h at room temperature. The slides were then washed with TBS and subjected to an additional 1 h of incubation with 10% goat serum. The secondary antibodies Alexa Fluor 555 goat anti-mouse (1:500, Invitrogen) and Alexa FluorPLUS 488 goat anti-rabbit (1:500, Invitrogen) were added for the visualization of CD138 and BCMA, respectively, with DAPI for 40 min at room temperature. The slides were washed with TBS and rinsed in water prior to being mounted with live cell media, coverslipped, and sealed for downstream imaging and technical analysis.

### 4.6. Imaging and Technical Analysis for Detection and Classification of Rare Cells

Image generation, technical analysis, and rare cell detection on the slide-based HDSCA immunofluorescence technology have been described previously [16,18,20,34]. Briefly, stained slides are imaged at 100× magnification, and an unsupervised clustering algorithm identifies rare cells based on cellular morphology and marker expression across all 2–3 million cells per slide, as described by 761 features per cell. Rare cells are segregated from common cells. The rare cell fraction from the automated algorithm is then classified based on CD138 and BCMA expression, and the candidate subtypes are visually inspected for cellular and morphological integrity, classified by channel type, and enumerated by a trained technician. The classifications of all reported cells are then reconfirmed by two additional researchers for reproducibility and cross-validation. The rare cell subtypes of interest as detected in this assay fall into the following primary categories:

- BCMA+CD138+CD45−
- BCMA+CD138−CD45−
- BCMA+CD138+CD45+
- BCMA−CD138+CD45−

While additional cellular and acellular events are detected and tracked by the OCULAR algorithm [16,17], this study focuses on the DAPI+ fraction indicated above. Enumeration results for each category are reported as the total count of cells per mL of blood.

### 4.7. Single-Cell Sequencing and CNV Analysis for Genomic Validation

To investigate whether the different rare cell subtypes harbor myeloma genomic alterations, and to delineate which subset of BCMA+ cells are aberrant, scCNV analysis was performed according to previously validated protocols [14,16]. In brief, a robotic micromanipulator was used to pick single cells off the glass slides and transfer them into PCR tubes containing lysis buffer (200 mM KOH; 50 mM DTT). The individual tubes were stored at −80 °C until the lysate was thawed and subjected to whole-genome amplification (WGA; Sigma-Aldrich, Burlington, MA, USA) and library construction using paired indices (Illumina). WGA was performed using the WGA4 Genomeplex Single-Cell Whole-Genome Amplification Kit (Sigma-Aldrich). Amplified DNA was purified using the QIAquick PCR purification kit (Qiagen, Hilden, Germany), and the resulting DNA was quantified using the Qubit Fluorometer (Thermo Fisher, Waltham, MA, USA). Indexed Illumina sequencing libraries were constructed and barcoded using the NEBNext Ultra DNA Library Preparation Kit (New England Biolabs, Ipswich, MA, USA). The amplified DNA fragments of the target size were sequenced at Fulgent Genomics (Temple City, CA, USA) to generate approximately 500,000 mapped reads. Sequenced reads were analyzed using our previously described CNV pipeline for reference genome mapping, read alignment, and single-cell ploidy determination [14,35,36]. For each sequenced and mapped cell, 2 copy numbers were normal, and any cell whose profile contained chromosomal locations with counts that deviate from the norm was considered to be harboring chromosomal alterations and, therefore, an aberrant cell. Chromosomal alterations were evaluated across the cells to establish the clonal relationships between cells from the same slide. WBCs (CD45+BCMA−CD138−) from each patient sample, along with cells of interest from ND samples, were included as negative controls and subjected to the same sequencing and analysis workflow. To evaluate whether BCMA-expressing cells were genomically altered, the morphological classification of sequenced cells was correlated with the scCNV profile status of altered or normal cells. Furthermore, BCMA expression was correlated with specific chromosomal events to see whether there were clonal cells in the early stages of B-cell differentiation and maturation. For initial clinical validation, the scCNV profiles of cells detected by our assay were compared to the patient-level cytogenetic alteration events probed in the MDACC’s targeted clinical FISH workup protocol as part of clinical diagnosis. For every single cell, we assessed the presence or absence of each cytogenetic event and quantified how many cells harbored genomic alterations observed using standard clinical methods.

### 4.8. Statistical Analysis

Data analysis was performed in the R programming language (version 3.6.3). Statistical comparison was computed using the Kruskal–Wallis test with a 95% confidence interval. The correlation between markers’ intensity was calculated using Pearson’s correlation, with the linear relationship reported using Pearson’s correlation coefficient.

## 5. Conclusions

This study reports the technical and initial clinical validation of an enrichment-free, slide-based single-cell immunofluorescence assay for the detection and molecular characterization of PCs in liquid biopsy samples collected from patients with MGUS, SMM, NDMM, RRMM, and LPL. Morphogenomic detection and characterization of normal and aberrant PCs provides insight into the heterogeneous BCMA expression and cellular localization. Genomic analysis of the scCNV profiles of circulating BCMA-expressing cells identified common genetic aberrations detected by conventional FISH and karyotyping in the standard clinical diagnosis and monitoring of PC cancers. Future large-scale validation studies are needed to demonstrate the clinical utility of this assay, with the potential to support clinical decision-making—especially in conditions associated with rare myeloma cells.

## Figures and Tables

**Table 1:** Patient demographics and clinical characteristics. FC: flow cytometry, sFLC: serum-free light chain, MGUS: monoclonal gammopathy of undetermined significance, SMM: smoldering multiple myeloma, NDMM: newly diagnosed multiple myeloma, RRMM: relapsed/refractory multiple myeloma, LPL: lymphoplasmacytic leukemia. Patient IDs are reflective of the disease state at diagnosis. VGPR: very good partial response.

	MGUS1	MGUS2	SMM1	SMM2	NDMM1	NDMM2	NDMM3	RRMM	LPL
**Age**	78	38	72	55	63	67	58	50	84
**Sex**	Male	Female	Female	Female	Female	Female	Female	Male	Male
**Diagnosis**	MGUS	MGUS	SMM	SMM	NDMM	NDMM	NDMM	RRMM	LPL
**Ig isotype**	IgG*k*	IgMλ	IgG*k*	IgG*k*	IgG*k*	IgA*k*	IgG*k*	IgDλ	IgGλ
**PCs in BM aspirate (%)**	1	4	10	10	15	76	7	27	2
**Aberrant PCs from the total PC BM compartment (%)**	92	0	78.9	98	95.2	100	99.6	99.9	94
**FC CD138**	Positive	NA	Positive	Positive	Positive	Positive	Positive	Positive	Positive
**FC CD38**	Positive	NA	Positive	Positive	Positive	Positive	Positive	Positive	Positive
**FC CD56**	Positive	NA	Positive	Negative	Positive	Positive	Positive	Positive	Negative
**FC CD45**	Negative	NA	Negative	Negative	Negative	Positive	Negative	Negative	Positive
**FC CD19**	Negative	NA	Negative	Negative	Negative	Negative	Negative	Negative	Positive
**FC CD27**	Positive	NA	Positive	Positive	Negative	Negative	Negative	Negative	Positive
**M-Spike (g/dL)**	0.7	0.4	3.9	1.5	0.4	0.2	2.1	0.3	0.2
**sFLC ratio**	8.14	1.06	37.58	8.45	186.84	1362.11	8.7	691.27	44.24
**Karyotype**	Normal	Normal	Normal	Normal	Normal	Abnormal *	Normal	Normal	Normal
**FISH**	Three copies of CCND1	Normal	Trisomy 11	Negative	Three copies of CCND1	t(4;14) and monosomy 13	Normal	t(11:14)	Normal
**Clinical presentation**	Low-risk MGUS for progression to MM by PETHEMA [21] criteria	Low-risk MGUS by PETHEMA criteria	High-risk SMM by PETHEMA criteria	High-risk SMM by PETHEMA criteria	Standard-risk NDMM	High-risk NDMM	Standard-risk NDMM	Standard-risk RRMM	Newly diagnosed Waldenström’s macroglobulinemia

* 43~45, X, del(X)(p22.1), +1, del(1)(p32), add(1)(p32), psu dic(3;1)(q25;p13), t(4;8) (q21;q24.3), −12, −13, der(14;18)(p10;q10), −22, +1~2 mar [cp16]/47, XX, +5[1]/46, XX[3].

**Table 2:** BCMA assay staining protocol. RT = room temperature; GS = goat serum.

Fixation: 2% PFA for 20 min
Wash: 1 × TBS 2 × 3 min
Blocking: 10% filtered GS in TBS for 30 min
Primary Mix:
CD138 mouse IgG1 (2 µg/mL)
BCMA rabbit IgG (2.5 µg/mL)
CD45-Alexa647 mouse IgG2a (1.6 µg/mL) incubated 1 h RT
Wash: 1 × TBS 2 × 3 min
10% filtered GS in TBS for 30 min
Secondary Mix: Alexa555 (1:500) + AlexaPLUS488 (1:500) + DAPI incubated 40 min RT
Wash and finish: 1 × TBS 2 × 3 min, dip ddH2O, coverslip with live cell media, seal

## Data Availability

All data discussed in this manuscript are included in the main manuscript text and the Supplementary Materials. The images of the single cells are available through the BloodPAC Data Commons Accession ID “BPDC000127” and the permalink https://data.bloodpac.org/discovery/BPDC000127/.

## Supplementary Materials

The supporting information can be downloaded at: https://www.mdpi.com/article/10.3390/ijms232113427/s1.

## References

[1] Banner D.W., D’Arcy A., Janes W., Gentz R., Schoenfeld H.-J., Broger C., Loetscher H., Lesslauer W. (1993). Crystal structure of the soluble human 55 kd TNF receptor-human TNFβ complex: Implications for TNF receptor activation. Cell.

[2] Madry C., Laabi Y., Callebaut I., Roussel J., Hatzoglou A., Le Coniat M., Mornon J.P., Berger R., Tsapis A. (1998). The characterization of murine BCMA gene defines it as a new member of the tumor necrosis factor receptor superfamily. Int. Immunol..

[3] Coquery C.M., Erickson L.D. (2012). Regulatory Roles of the Tumor Necrosis Factor Receptor BCMA. Crit. Rev. Immunol..

[4] Gras M.-P., Laâbi Y., Linares-Cruz G., Blondel M.-O., Rigaut J.-P., Brouet J.-C., Haguenauer-Tsapis R., Leca G., Tsapis A. (1995). BCMAp: An integral membrane protein in the Golgi apparatus of human mature B lymphocytes. Int. Immunol..

[5] Hatzoglou A., Roussel J., Bourgeade M.-F., Rogier E., Madry C., Inoue J., Devergne O., Tsapis A. (2000). TNF Receptor Family Member BCMA (B Cell Maturation) Associates with TNF Receptor-Associated Factor (TRAF) 1, TRAF2, and TRAF3 and Activates NF-κB, Elk-1, c-Jun N-Terminal Kinase, and p38 Mitogen-Activated Protein Kinase. J. Immunol..

[6] Ryan M.C., Hering M., Peckham D., McDonagh C.F., Brown L., Kim K.M., Meyer D.L., Zabinski R.F., Grewal I.S., Carter P.J. (2007). Antibody targeting of B-cell maturation antigen on malignant plasma cells. Mol. Cancer Ther..

[7] Cho S.-F., Anderson K.C., Tai Y.-T. (2018). Targeting B Cell Maturation Antigen (BCMA) in Multiple Myeloma: Potential Uses of BCMA-Based Immunotherapy. Front. Immunol..

[8] Dogan A., Siegel D., Tran N., Fu A., Fowler J., Belani R., Landgren O. (2020). B-cell maturation antigen expression across hematologic cancers: A systematic literature review. Blood Cancer J..

[9] Ware C.F. (2011). The TNF receptor super family in immune regulation. Immunol. Rev..

[10] Sanchez L., Dardac A., Madduri D., Richard S., Richter J. (2021). B-cell maturation antigen (BCMA) in multiple myeloma: The new frontier of targeted therapies. Ther. Adv. Hematol..

[11] Shah N., Chari A., Scott E., Mezzi K., Usmani S.Z. (2020). B-cell maturation antigen (BCMA) in multiple myeloma: Rationale for targeting and current therapeutic approaches. Leukemia.

[12] Salem D.A., Maric I., Yuan C.M., Liewehr D.J., Venzon D.J., Kochenderfer J., Stetler-Stevenson M. (2018). Quantification of B-cell maturation antigen, a target for novel chimeric antigen receptor T-cell therapy in Myeloma. Leuk. Res..

[13] Frigyesi I., Adolfsson J., Ali M., Christophersen M.K., Johnsson E., Turesson I., Gullberg U., Hansson M., Nilsson B. (2014). Robust isolation of malignant plasma cells in multiple myeloma. Blood.

[14] Welter L., Xu L., McKinley D., Dago A.E., Prabakar R.K., Restrepo-Vassalli S., Xu K., Rodriguez-Lee M., Kolatkar A., Nevarez R. (2020). Treatment response and tumor evolution: Lessons from an extended series of multianalyte liquid biopsies in a metastatic breast cancer patient. Cold Spring Harb. Mol. Case Stud..

[15] Ruiz C., Li J., Luttgen M.S., Kolatkar A., Kendall J.T., Flores E., Topp Z., E Samlowski W., McClay E., Bethel K. (2015). Limited genomic heterogeneity of circulating melanoma cells in advanced stage patients. Phys. Biol..

[16] Chai S., Matsumoto N., Storgard R., Peng C.-C., Aparicio A., Ormseth B., Rappard K., Cunningham K., Kolatkar A., Nevarez R. (2021). Platelet-Coated Circulating Tumor Cells Are a Predictive Biomarker in Patients with Metastatic Castrate-Resistant Prostate Cancer. Mol. Cancer Res..

[17] Shishido S.N., Sayeed S., Courcoubetis G., Djaladat H., Miranda G., Pienta K.J., Nieva J., Hansel D.E., Desai M., Gill I.S. (2022). Characterization of Cellular and Acellular Analytes from Pre-Cystectomy Liquid Biopsies in Patients Newly Diagnosed with Primary Bladder Cancer. Cancers.

[18] Kolenčík D., Narayan S., Thiele J.-A., McKinley D., Gerdtsson A.S., Welter L., Hošek P., Ostašov P., Vyčítal O., Brůha J. (2022). Circulating Tumor Cell Kinetics and Morphology from the Liquid Biopsy Predict Disease Progression in Patients with Metastatic Colorectal Cancer Following Resection. Cancers.

[19] Zhang L., Beasley S., Prigozhina N.L., Higgins R., Ikeda S., Lee F.Y., Marrinucci D., Jia S. (2016). Detection and Characterization of Circulating Tumour Cells in Multiple Myeloma. J. Circ. Biomarkers.

[20] Ndacayisaba L.J., Rappard K.E., Shishido S.N., Velasco C.R., Matsumoto N., Navarez R., Tang G., Lin P., Setayesh S.M., Naghdloo A. (2022). Enrichment-Free Single-Cell Detection and Morphogenomic Profiling of Myeloma Patient Samples to Delineate Circulating Rare Plasma Cell Clones. Curr. Oncol..

[21] Pérez-Persona E., Vidriales M.-B., Mateo G., García-Sanz R., Mateos M.-V., de Coca A.G., Galende J., Martín-Nuñez G., Alonso J.M., de Las Heras N. (2007). New Criteria to Identify Risk of Progression in Monoclonal Gammopathy of Uncertain Significance and Smoldering Multiple Myeloma Based on Multiparameter Flow Cytometry Analysis of Bone Marrow Plasma Cells. Blood.

[22] Laurent S.A., Hoffmann F.S., Kuhn P.-H., Cheng Q., Chu Y., Schmidt-Supprian M., Hauck S., Schuh E., Krumbholz M., Rübsamen H. (2015). γ-secretase directly sheds the survival receptor BCMA from plasma cells. Nat. Commun..

[23] Bujarski S., Soof C., Chen H., Li M., Sanchez E., Wang C.S., Emamy-Sadr M., Swift R.A., Rahbari K.J., Patil S. (2018). Serum b-cell maturation antigen levels to predict progression free survival and responses among relapsed or refractory multiple myeloma patients treated on the phase I IRUX trial. J. Clin. Oncol..

[24] Visram A., Soof C., Rajkumar S.V., Kumar S.K., Bujarski S., Spektor T.M., Kyle R.A., Berenson J.R., Dispenzieri A. (2021). Serum BCMA levels predict outcomes in MGUS and smoldering myeloma patients. Blood Cancer J..

[25] Secretase Inhibition Increases Efficacy of BCMA-Specific Chimeric Antigen Receptor T Cells in Multiple Myeloma|Blood|American Society of Hematology. https://ashpublications.org/blood/article/134/19/1585/374996/Secretase-inhibition-increases-efficacy-of-BCMA.

[26] Johnsen H.E., Bøgsted M., Schmitz A., Bødker J.S., El-Galaly T.C., Johansen P., Valent P., Zojer N., Van Valckenborgh E., Vanderkerken K. (2016). The myeloma stem cell concept, revisited: From phenomenology to operational terms. Haematologica.

[27] Gao M., Bai H., Jethava Y., Wu Y., Zhu Y., Yang Y., Xia J., Cao H., Franqui-Machin R., Nadiminti K. (2019). Identification and Characterization of Tumor-Initiating Cells in Multiple Myeloma. JNCI J. Natl. Cancer Inst..

[28] Smith E.L., Harrington K., Staehr M., Masakayan R., Jones J., Long T.J., Ng K.Y., Ghoddusi M., Purdon T.J., Wang X. (2019). GPRC5D is a target for the immunotherapy of multiple myeloma with rationally designed CAR T cells. Sci. Transl. Med..

[29] Fernández de Larrea C., Staehr M., Lopez A.V., Ng K.Y., Chen Y., Godfrey W.D., Purdon T.J., Ponomarev V., Wendel H.-G., Brentjens R.J. (2020). Defining an Optimal Dual-Targeted CAR T-cell Therapy Approach Simultaneously Targeting BCMA and GPRC5D to Prevent BCMA Escape–Driven Relapse in Multiple Myeloma. Blood Cancer Discov..

[30] Elkins K., Zheng B., Go M., Slaga D., Du C., Scales S.J., Yu S.-F., McBride J., de Tute R., Rawstron A. (2012). FcRL5 as a Target of Antibody–Drug Conjugates for the Treatment of Multiple Myeloma. Mol. Cancer Ther..

[31] Stewart A.K., Krishnan A.Y., Singhal S., Boccia R.V., Patel M.R., Niesvizky R., Chanan-Khan A.A., Ailawadhi S., Brumm J., Mundt K.E. (2019). Phase I study of the anti-FcRH5 antibody-drug conjugate DFRF4539A in relapsed or refractory multiple myeloma. Blood Cancer J..

[32] Ray A., Song Y., Du T., Buon L., Tai Y.-T., Chauhan D., Anderson K.C. (2022). Identification and validation of ecto-5′ nucleotidase as an immunotherapeutic target in multiple myeloma. Blood Cancer J..

[33] Leow C., Low M. (2021). Targeted Therapies for Multiple Myeloma. J. Pers. Med..

[34] Shishido S.N., Welter L., Rodriguez-Lee M., Kolatkar A., Xu L., Ruiz C., Gerdtsson A.S., Restrepo-Vassalli S., Carlsson A., Larsen J. (2020). Preanalytical Variables for the Genomic Assessment of the Cellular and Acellular Fractions of the Liquid Biopsy in a Cohort of Breast Cancer Patients. J. Mol. Diagn..

[35] Baslan T., Kendall J., Ward B., Cox H., Leotta A., Rodgers L., Riggs M., D’Italia S., Sun G., Yong M. (2015). Optimizing sparse sequencing of single cells for highly multiplex copy number profiling. Genome Res..

[36] Dago A.E., Stepansky A., Carlsson A., Luttgen M., Kendall J., Baslan T., Kolatkar A., Wigler M., Bethel K., Gross M.E. (2014). Rapid Phenotypic and Genomic Change in Response to Therapeutic Pressure in Prostate Cancer Inferred by High Content Analysis of Single Circulating Tumor Cells. PLoS ONE.

