# Supplementary material for: Characterization of BCMA Expression in Circulating Rare Single Cells of Patients with Plasma Cell Neoplasms
Source: Int J Mol Sci. 2022 Nov 3;23(21):13427. doi: 10.3390/ijms232113427 (PMC9658574; doi:10.3390/ijms232113427)
Supplement: Supplementary file 1 [file ijms-23-13427-s001.zip › ijms-1960236-supplementary.pdf]

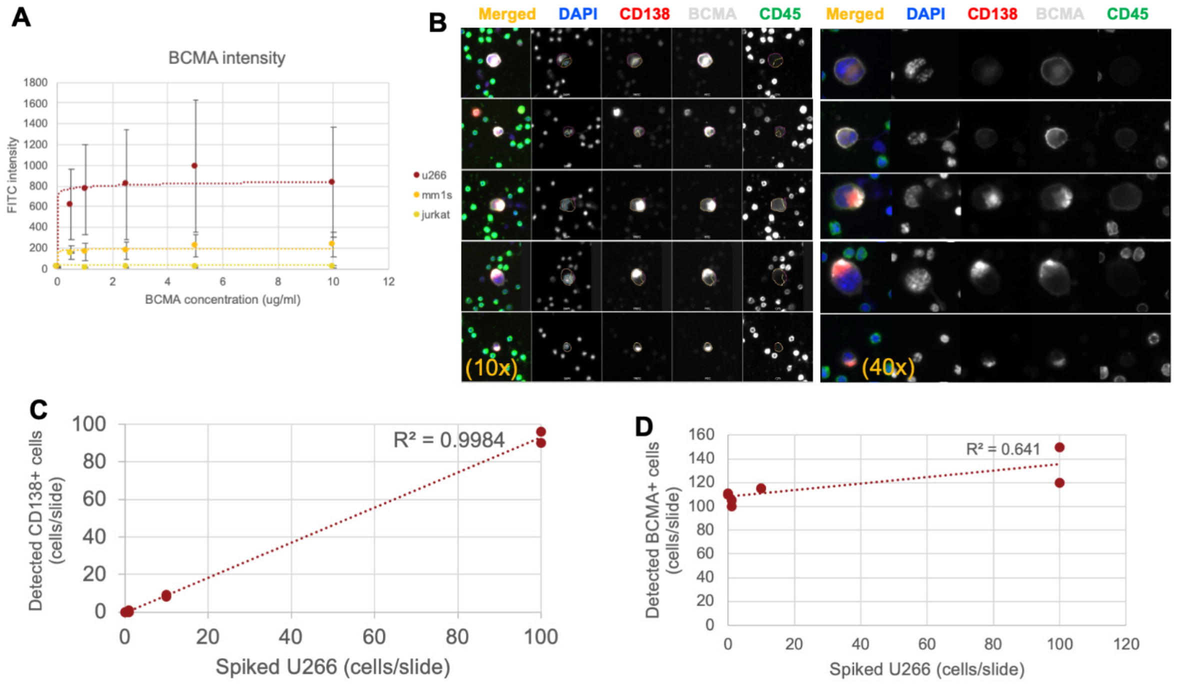

Figure S1: Assay development and validation in cell lines and spiked normal blood. **(A)** Titration curve of BCMA at 0-10µg/mL concentrations in U266, MM.1S, and Jurkat cell lines. (**B)** Representative images at 100x and 400x magnification of BCMA+ U266 cells with surrounding WBCs (green). (**C)** Linearity between cell counts in spiked-in experiments for CD138+ cells. (**D)** Linearity for BCMA+ cells in U266 spiked in NBD.

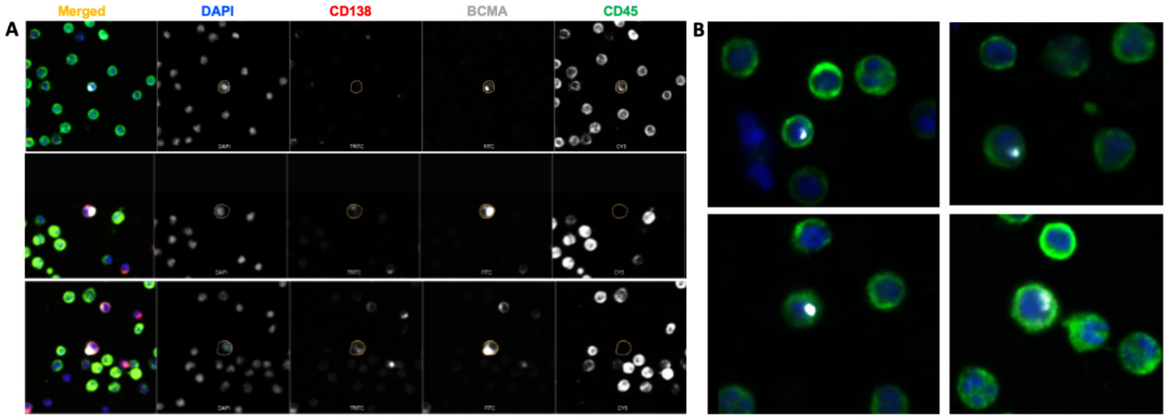

Figure S2: Representative cells from high content microscopy imaging of observed staining pattern consistent with previously reported BCMAp. (A) 100x magnification. **(B)** 400x magnification
